# Risk factors for early radiation-induced heart damage in patients undergoing pulmonary SBRT

**DOI:** 10.1186/s44156-025-00076-1

**Published:** 2025-06-02

**Authors:** Tingcui Li, Dan Zhu, Ming Cui

**Affiliations:** 1 Department of Intensive Care Unit, Peking University Third Hospital, Beijing, China; 2 Department of Cardiology, Peking University Third Hospital, Beijing, China

**Keywords:** Radiation-induced heart damage, Cardiotoxicity, Stereotactic body radiotherapy, Global longitudinal strain, Risk factors

## Abstract

**Background:**

Stereotactic body radiotherapy (SBRT) is superior to conventional radiotherapy for the treatment of lung tumors but can lead to radiation-induced heart damage (RIHD). Its risk factors have not been clarified. The purpose of our study was to determine the risk factors for early RIHD in patients undergoing pulmonary SBRT.

**Methods:**

We prospectively included patients who planned to receive pulmonary SBRT at our center from January 2020 to May 2021. Two-dimensional speckle tracking echocardiography was performed within 2 months after radiotherapy. The diagnostic criterion for early RIHD was a decrease in global longitudinal strain by ≥ 15% from baseline. Logistic regression was used to explore the risk factors for early RIHD.

**Results:**

A total of 108 patients were included in the study. The overall incidence of early RIHD in the cohort was 41.7%. Significant risk factors, including maximum heart dose, anthracycline use and hypertension, were independently associated with early RIHD, with ORs of 1.058 (95% CI: 1.028–1.089; *p* < 0.001), 3.524 (95% CI: 1.296–9.577; *p* = 0.014), and 4.284 (95% CI: 1.424–12.890; *p* = 0.010), respectively. The cutoff of the maximum heart dose was 27.0 Gy in patients who received anthracycline and 29.3 Gy in those who did not.

**Conclusions:**

Among patients receiving pulmonary SBRT, the maximum heart radiation dose, the use of anthracycline drugs and hypertension are independently associated with the occurrence of early RIHD. These findings could be applied to predict early RIHD and screen for high-risk patients. Individualized cardiac dose limitations may be helpful in improving the long-term prognosis of pulmonary SBRT patients.

**Supplementary Information:**

The online version contains supplementary material available at 10.1186/s44156-025-00076-1.

## Introduction

Radiation-induced heart damage (RIHD) refers to a series of structural and functional changes in the heart caused by radiation exposure. It mainly occurs in patients who have undergone transthoracic radiotherapy; these changes involved lung cancer, lung metastasis, lymphoma, breast cancer, and esophageal cancer [1]. Compared with patients who did not receive radiotherapy, patients who received transthoracic radiotherapy had a 27% higher risk of cardiac-related death [2]. In patients with non-small-cell lung cancer, the risk of adverse cardiac events increased significantly within 2 years after radiotherapy due to receiving higher cardiac dosages, resulting decreased overall survival. This in turn, leads to reduction in the survival benefits of radiotherapy [3]. RIHD can affect any structure of the heart and has become one of the major complications threatening the survival of cancer patients after radiotherapy [4, 5].

Stereotactic body radiotherapy (SBRT) is a precision radiotherapy technology with a high fractionated dose. Its fractionated dose can reach more than 10 Gy, with better treatment effect on lung cancer than conventional radiotherapy [6]. Therefore, radiotherapy has become one of the main methods for treating pulmonary tumor [7]. Though pulmonary SBRT can significantly reduce the overall dose to the heart, it can also expose part of the heart to a higher local dose, increasing the risk of long-term adverse cardiovascular events [8, 9]. Under an equivalent total dose of radiotherapy, the damage to the heart caused by a high fractionated dose is more significant than that caused by a low fractionated dose [10]. At present, the risk factors for RIHD caused by pulmonary SBRT are unclear.

The purpose of this study was to determine the risk factors related to early RIHD in patients receiving lung SBRT to improve long-term prognosis of patients with lung cancer and to reduce their risk of adverse cardiovascular events.

## Methods

### Study oversight and design

This was an investigator-initiated, single center, prospective cohort study conducted at Peking University Third Hospital. The study was approved by the Medical Science Research Ethics Committee of Peking University Third Hospital (NCT04443400, registration date: 21 June 2020). Patients included in the study signed informed consent forms. All procedures performed in studies involving human participants were in accordance with the ethical standards of the institutional research committee. This trial was conducted in compliance with the 1964 Helsinki Declaration and its later amendments.

### Patients

From January 2020 to April 2021, 108 patients who met the clinical criteria for malignant tumors before receiving pulmonary SBRT at our hospital were enrolled in the study after being screened for eligibility. The inclusion criteria were: (1) patients with indications for radiotherapy who will receive pulmonary SBRT, (2) cardiac exposure to radiation during pulmonary SBRT, and (3) left ventricular ejection fraction (LVEF) of > 50%. The exclusion criteria were: (1) patients without satisfactory echocardiographic images (2) patients with acute coronary syndrome, heart failure (NYHA III-IV), heart valve disease (moderate and severe valve regurgitation and/or stenosis), arrhythmias requiring intervention (requiring medication, radiofrequency ablation or pacemaker treatment), congenital heart disease, cardiomyopathy, pericardial effusion, pulmonary artery systolic pressure ≥ 50mmHg, severe liver and kidney dysfunction (alanine aminotransferase and/or aspartate aminotransferase ≥ 3 times the upper limit of reference value, creatinine > 130 mol/L), and autoimmune diseases, and (3) patients who participated in other clinical studies.

### Follow-up and RIHD assessment

All patients were scheduled for clinical follow-up at site visits within 2 months of SBRT. Echocardiography, two-dimensional speckle tracking echocardiography (2D-STE), and measurements of CK, CK-MB, cTnT and NT-proBNP were performed before and after SBRT. RIHD was defined as at least a 15% reduction of global longitudinal strain (GLS) from baseline after SBRT.

### Echocardiography and 2D-STE

Echo-Doppler data were obtained using a GE Vivid E9 ultrasonography system. LVEF was calculated by the modified Simpson or modified Quinones method. Left ventricular mass (LVM) was calculated by the Devereux formula and indexed for body surface area. Left ventricular strain analysis was performed from stored transthoracic echocardiography images using a TomTec Imaging System. Patients were excluded from the analysis if more than 3 segments were deemed unsatisfactory.

### Stereotactic body radiotherapy

Chest CT was carried out to locate the target area before SBRT. The gross tumor volume (GTV) was delineated according to the location on CT. The planned target volume (PTV) was moderately extended by 3–5 mm based on the GTV. The treatment plan was formulated using the Multiplan 4.6 planning system. The minimum, average, and maximum doses to the heart and the V5 and V10 doses to the heart were determined according to the treatment plan. The biologically effective dose (BED) and equivalent dose in 2-Gy fractions (EQD2) were calculated according to the prescribed dose and fraction [11, 12]. BED = nd[1 + d/(α/β)], EQD2 = nd[(d + α/β)/(2 + α/β)], α/β = 3 Gy, n is the number of fractions, and d is the dose per fraction [13].

### Statistical analysis

The measurement data were tested for normality with the Kolmogorov-Smirnov test. Normally distributed data are expressed as mean ± standard deviation. Nonnormally distributed data are reported as frequencies and percentages. Subjects were classified in two groups (RIHD vs. non-RIHD) based on whether RIHD occurred within 2 months after SBRT. The independent-sample t-test, chi-square test, Fisher’s exact test or Mann-Whiney U test was used for inter-group comparison, as appropriate. The paired sample t-test or Wilcoxon signed rank test was used for intra-group comparison. Logistic regression analysis was used to identify risk factors for RIHD. *p* < 0.05 was considered significant. Statistical analyses were performed with SPSS 22.0.

## Results

### Baseline characteristics of the study population

**Fig. 1:**
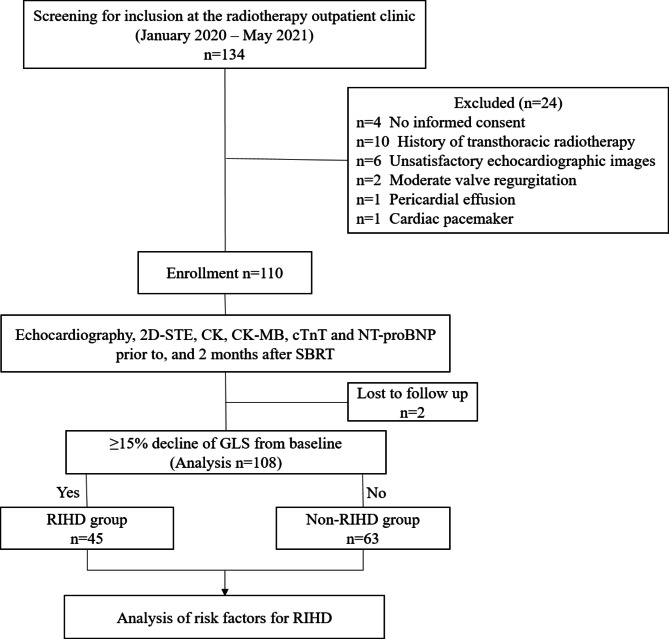
Trial profile. 2D-STE = two-dimensional speckle tracking echocardiography; SBRT = Stereotactic body radiotherapy; RIHD = Radiation-induced heart damage

A total of 110 participants were included in this study. Two of 110 participants were lost to follow-up, with a total of 108 participants completing the 2-month follow-up. The changes in cardiac damage indicators before and after SBRT are shown in eTable 1 (Supplementary Appendix). Among the 108 participants, 45 (42%) developed early RIHD and 63 (58%) did not. The incidence rate of early RIHD within 2 months after receiving pulmonary SBRT was 41.7% (Fig. 1).

The incidence of hypertension was higher in the RIHD subgroup than in the non-RIHD subgroup (15 [33%] of 45 patients vs. 9 [14%] of 63 patients; *p* = 0.019). LVM was lower in the RIHD group than in the non-RIHD group. The E/Em ratio and left atrial pressure were significantly higher in the RIHD group than in the non-RIHD group. We found no difference in baseline myocardial strain parameters, including global longitudinal strain, global circumferential strain or global radial strain, between the two groups. The baseline characteristics of the study population are shown in Table 1.

**Table 1:** Patient characteristics

	RIHD group (*n* = 45)	Non-RIHD group (*n* = 63)	*p* value
Age, in years	56.0(28.5, 74.0)	56.0(38.0, 70.0)	0.89
Male sex, n (%)	23(51.1)	28(44.4)	0.49
BMI, kg/m2	24.3 ± 5.1	23.0 ± 4.3	0.15
Systolic pressure, mmHg	129.0(120.0, 136.5)	124.0(116.0,134.6)	0.19
Diastolic pressure, mmHg	75.2 ± 8.9	75.1 ± 8.4	0.96
Heart rate, times/min	82.9 ± 16.3	78.7 ± 14.3	0.15
CAD, no. (%)	3(6.7)	5(7.9)	1.00
Hypertension, no. (%)	15(33.3)	9(14.3)	0.019*
Diabetes, no. (%)	8(17.8)	6(9.5)	0.21
Dyslipidemia, no. (%)	20(44.4)	27(42.9)	0.87
Smoking, no. (%)	7(15.6)	15(23.8)	0.29
ACEI/ARB, n (%)	7(15.6)	3(4.8)	0.09
β receptor blocker, n (%)	4(8.9)	2(3.2)	0.23
Statins, n (%)	4(8.9)	7(11.1)	0.76
FBG, mmol/L	5.7(5.3, 6.5)	5.3(5.0, 5.8)	0.033*
TC, mmol/L	4.7(3.7, 5.2)	4.5(3.9, 5.6)	0.77
TG, mmol/L	1.6(1.0, 2.3)	1.4(1.1, 1.8)	0.42
LDL-C, mmol/L	2.8(2.3, 3.3)	2.7(2.3, 3.7)	0.59
HDL-C, mmol/L	1.2 ± 0.3	1.2 ± 0.3	0.42
CKMB, U/L	9.0(6.0, 16.0)	9.0(6.0, 12.0)	0.32
TnT, ng/mL	0.010(0.006, 0.013)	0.007(0.004, 0.011)	0.091
NT-proBNP, pg/mL	95.0(39.0, 159.5)	65.0(20.0, 108.0)	0.19
LVEF, %	69.0(64.5, 72.0)	70.0(68.0, 72.0)	0.2
LVM, g	104.0 ± 28.4	119.7 ± 33.2	0.012*
E/A	0.9(0.8, 1.1)	0.8(0.7, 1.2)	0.22
E/Em	7.0(5.0, 10.0)	6.0(5.0, 7.0)	0.009*
LAP, mmHg	10.0(8.0, 14.0)	9.0(8.0, 10.0)	0.009*
GLS, %	-11.9 ± 4.0	-12.8 ± 4.2	0.23
GCS, %	-12.9 ± 4.5	-13.1 ± 5.1	0.84
GRS, %	19.1 ± 12.5	19.5 ± 12.6	0.87

Values are mean ± SD, n (%), or median (interquartile range). The p values refer to the difference between RIHD group and non-RIHD group. *p < 0.05. BMI = body mass index; CAD = coronary artery disease; ACEI = angiotensin converting enzyme inhibitor; ARB = angiotensin receptor blocker; FBG = fasting blood glucose; TC = total cholesterol; TG = triglyceride; CKMB = creatine kinase isoenzyme; TnT = troponin T; NT-proBNP = N-terminal pro-B-type natriuretic peptide; LVEF = left ventricular ejection fraction; LVM = left ventricular mass; LAP = left atrial pressure; GLS = global longitudinal strain; GCS = global circumferential strain; GRS = global radial strain

### Tumor characteristics and antitumor drug use

There was no significant difference in tumor type or tumor stage between the two groups. At baseline, more patients took anthracyclines in the RIHD group than the non-RIHD group. There were no significant differences between the groups regarding history of chemotherapy, targeted drugs or immune checkpoint inhibitor use (Table 2).

**Table 2:** Tumor characteristics and antitumor drug use

	RIHD group (*n* = 45)	Non-RIHD group (*n* = 63)	*p* value
Tumor classification, n (%)	-	-	0.6
Lung cancer	22(48.9)	34(54.0)	-
Pulmonary metastasis tumor	23(51.1)	29(46.0)	-
Tumor Stage, no. (%)			0.89
I	13(28.9)	14(22.2)	-
II	1(2.2)	1(1.6)	-
III	1(2.2)	2(3.2)	-
IV	30(66.7)	46(73.0)	-
Medication, no. (%)			
Chemotherapy history	30(66.7)	34(54.0)	0.19
Anthracycline	21(46.7)	15(23.8)	0.013*
Targeted drugs	27(60.0)	26(41.3)	0.055
Immune checkpoint inhibitors	12(26.7)	10(15.9)	0.17

Values are n (%). * *p* < 0.05.

### Stereotactic body radiotherapy data

Our analysis revealed no statistically significant differences between the two groups in radiotherapy location, prescription dose, fractionation, BED, EQD2, PTV or GTV (*p* > 0.05 for all). The indices related to cardiac dose, including the minimum heart dose, mean heart dose, maximum heart dose, V5 and V10, were significantly higher in the RIHD group (Table 3).

**Table 3:** Stereotactic body radiotherapy characteristics and parameters

	RIHD group (*n* = 45)	Non-RIHD group (*n* = 63)	*p* value
Radiotherapy location, n (%)			0.37
Upper lobe of left lung	10(22.2)	13(20.6)	-
Lower lobe of left lung	4(8.9)	16(25.4)	-
Upper lobe of right lung	7(15.6)	9(14.3)	-
Middle lobe of right lung	2(4.4)	2(3.2)	-
Lower lobe of right lung	5(11.1)	6(9.5)	-
Multiple lung lobes	17(37.8)	17(27.0)	-
Prescription dose, Gy	40.0(33.0, 45.0)	36.0(30.0, 45.0)	0.57
Fractionation, times	3.0(3.0, 5.0)	3.0(3.0, 3.0)	0.11
BED, Gy	180.0(143.5, 270.0)	180.0(147.0, 270.0)	0.26
EQD2, Gy	108.0(88.0, 162.0)	108.0(88.0, 162.0)	0.29
PTV, cm^3^	28.9(8.0, 88.9)	18.2(8.3, 59.0)	0.29
GTV, cm^3^	7.4(0.8, 37.0)	3.6(0.7, 17.0)	0.16
Minimum heart dose, Gy	0.6(0.3, 1.1)	0.4(0.2, 0.7)	0.017*
MHD, Gy	6.8(3.1, 10.0)	2.2(1.2, 6.2)	< 0.001*
Maximum heart dose, Gy	36.6(18.2, 48.8)	15.6(8.3, 25.3)	< 0.001*
V5, %	50.5(18.2, 72.4)	12.6(2.3, 52.2)	0.001*
V10, %	23.7(2.4, 35.9)	1.3(0, 20.1)	0.001*

Values are n (%), or median (interquartile range). BED = biologically effective dose; EQD2 = equivalent dose in 2 Gy per fraction; PTV = planned target volume; GTV = gross tumor volume; MHD = mean heart dose; V5 and V10 = percentages of heart volume receiving ≥ 5 Gy and ≥ 10 Gy. * *p* < 0.05.

### Risk factors for early radiation-induced heart damage

The risk factors for early RIHD caused by SBRT are not yet clear. In the univariate analysis, we included age, gender, maximum cardiac dose, complication (hypertension, diabetes mellitus and coronary heart disease), medication history (anthracyclines, targeted drugs, immunosuppressive agents, angiotensin converting enzyme inhibitor/angiotensin receptor blocker, β blockers and statins), smoking history, TnT, NT-proBNP, LVM, LVEF, E/A, E/Em, left atrial pressure and GLS into the logistic regression model, and carried out binary Logistic regression analysis with early RIHD as the dependent variable. The results showed that maximum cardiac dose, use of anthracyclines, hypertension, LVM, E/Em, and LAP were associated with the occurrence of early RIHD (Supplementary Appendix eTable 1).

Then, we performed a multivariate analysis to determine the risk factors for early RIHD. A total of five indicators were included in the multivariate logistic regression model: the maximum heart dose, anthracycline use, hypertension, LVM, and E/Em. We ran a binary logistic regression with early RIHD occurrence as the dependent variable. The entry method was forward (LR), the entry criterion was *p* < 0.05, and the exclusion criterion was *p* > 0.1. We found that the maximum heart dose (OR = 1.058; 95% CI: 1.028–1.089; *p* < 0.001), history of anthracycline use (OR = 3.524, 95% CI: 1.296–9.577; *p* = 0.014) and history of hypertension (OR = 4.284; 95% CI: 1.424–12.890; *p* = 0.010) were independent risk factors for early RIHD (Table 4).

**Table 4:** Risk factors of early RIHD

	***B***	S.E.	Odds Ratio (95%CI)	*p* value
Maximum heart dose, Gy	0.057	0.015	1.058(1.028, 1.089)	< 0.001*
Anthracycline, n (%)	1.260	0.510	3.524(1.296, 9.577)	0.014*
Hypertension, n (%)	1.460	0.565	4.284(1.424, 12.890)	0.010*
LVM, g	-	-	-	0.23
E/Em	-	-	-	0.068

LVM = left ventricular mass. *p < 0.05.

**Fig. 2:**
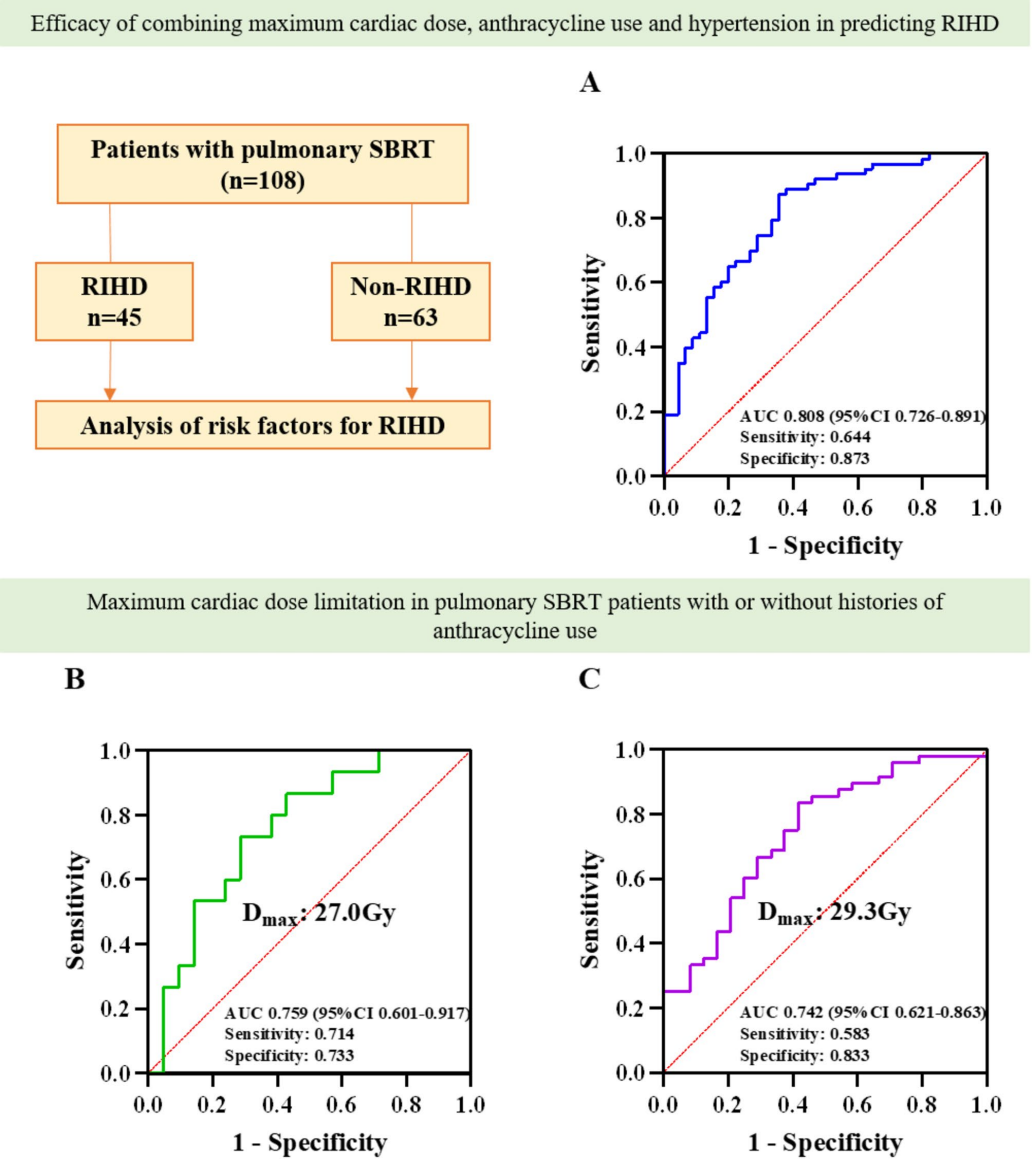
**Risk factors of early RIHD in patients with pulmonary SBRT.** The maximum cardiac dose, history of anthracycline use, and hypertension are related to the occurrence of early RIHD in patients with pulmonary SBRT. Patients with or without histories of anthracyclines use have different maximum cardiac limiting doses. **A**: Combined predictive value of maximum cardiac dose, anthracycline use, and hypertension for early RIHD; **B**: Maximum cardiac limiting dose for patients with histories of anthracyclines use; **C**: Maximum cardiac limiting dose for patients without histories of anthracyclines use. RIHD = Radiation-induced heart damage; SBRT = Stereotactic body radiotherapy; D_max_: Maximum heart dose

To evaluate the predictive value of the regression model for early RIHD, we plotted a receiver operating characteristic (ROC) curve for predicting early RIHD using the maximum cardiac dose, anthracycline use, and hypertension. The results showed that the area under the ROC curve was 0.808 (95% CI 0.726 to 0.891, *p* < 0.001), with a sensitivity of 64.4% and a specificity of 87.3%, as shown in Fig. 2 (A).

Radiotherapy combined with chemotherapy plays an important role in tumor treatment, and radiotherapy combined with chemotherapy may further increase the occurrence of early RIHD. Individualized treatment may improve the prognosis of these patients. We further analyzed the cardiac dose limit of pulmonary SBRT in patients with a history of anthracycline use. Fig. 2 (B and C) show the ROC curves. A maximum heart dose of 27.0 Gy (AUC = 0.759, *p* = 0.009) in patients with a history of anthracycline use and 29.3 Gy (AUC = 0.742, *p* = 0.001) in patients without a history of anthracycline use all predicted the occurrence of early RIHD after pulmonary SBRT.

### Case report

#### Patient 1

**Fig. 3:**
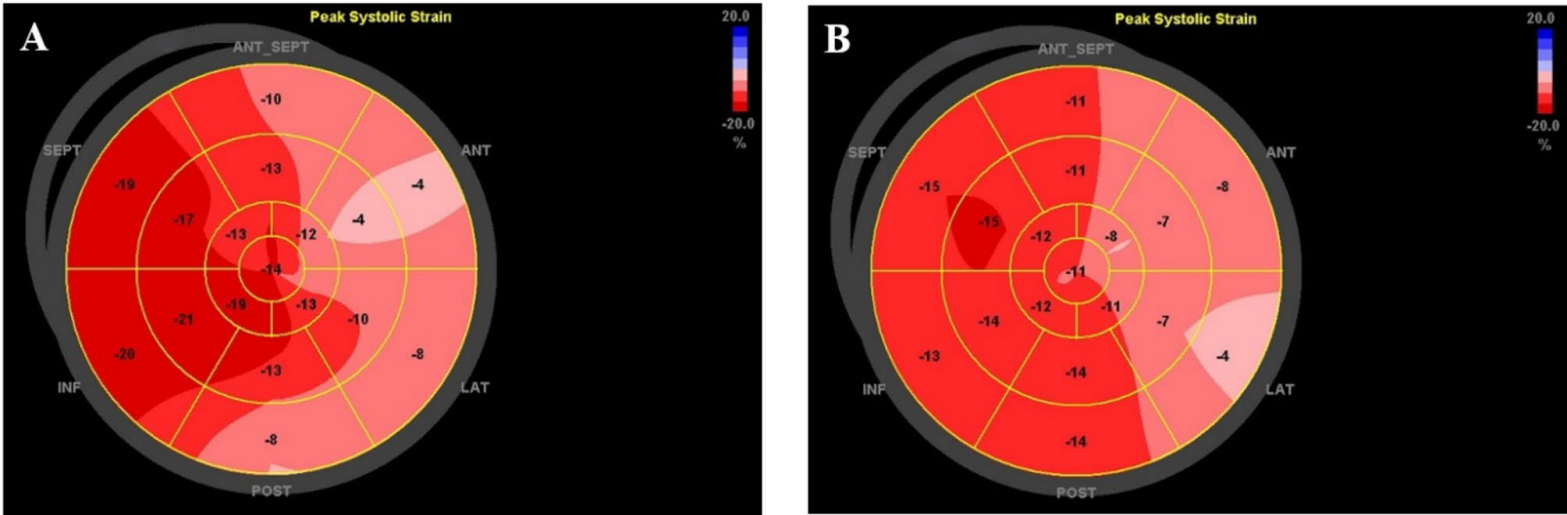
**Changes in bull’s eye plot in patients with a history of anthracyclines exposure. **The polar map derived by 2D-STE shows the bull’s eye plots in a patient with a history of anthracycline therapy before and after radiotherapy. **A**: Baseline bull’s eye plot before SBRT; **B**: Bull’s eye plot 2 months after SBRT

A 48-year-old female with a history of rhabdomyosarcoma and lung metastasis (exposed to anthracyclines and targeted therapy before radiotherapy, without other cardiovascular diseases) underwent monitoring for cardiotoxicity during lung SBRT. Baseline echocardiography showed an LVEF of 0.7, and STE analysis revealed a GLS of -12.7%. Two months later, there was no significant change in the patient’s left ventricular ejection fraction, but her GLS decreased to -10.8% (Fig. 3).

### Patient 2

**Fig. 4:**
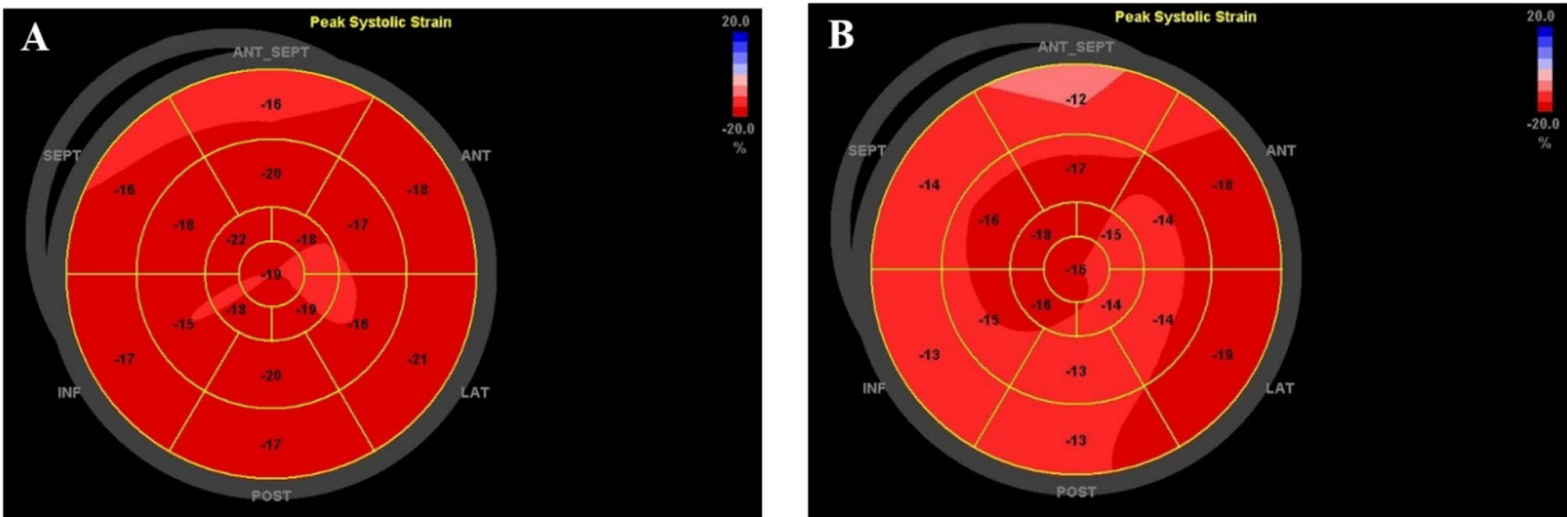
**Changes in bull’s eye plot in patients without a history of anthracyclines exposure.** The polar map derived by 2D-STE shows the bull’s eye plots in a patient without a history of anthracycline therapy before and after radiotherapy. **A**: Baseline bull’s eye plot before SBRT; **B**: Bull’s eye plot 2 months after SBRT

A 72-year-old female with a history of lung adenocarcinoma (no exposure to anthracyclines before radiotherapy, but with a history of targeted therapy, without other cardiovascular diseases) underwent monitoring for cardiotoxicity during lung SBRT. Baseline echocardiography showed an LVEF of 0.67, and STE analysis revealed a GLS of -18.2%. Two months later, there was no significant change in the patient’s left ventricular ejection fraction, but her GLS decreased to -14.9% (Fig. 4).

## Discussion

In this study, 2D-STE was used to evaluate the cardiotoxicity caused by pulmonary SBRT. The results of this study highlight the risk factors for pulmonary SBRT and suggest the appropriate maximum heart dose for patients with a history of anthracycline use or without a history of anthracycline use. Overall, we have made three important findings in lung tumor patients. First, the overall incidence of early RIHD after pulmonary SBRT was 41.7%. Second, the maximum heart dose of SBRT, the use of anthracycline drugs and the presence of hypertension were independently associated with the occurrence of early RIHD. The AUC of their combination for predicting early RIHD was 0.808, the sensitivity was 64.4%, and the specificity was 87.3%. Third, the cutoff value of the maximum heart dose was 27.0 Gy in patients with a history of anthracycline use and 29.3 Gy in patients without a history of anthracycline use.

2D-STE can be used to evaluate cardiac function in three different directions—longitudinal, circumferential and radial—without angle dependence and can accurately reflect myocardial movement. Its main advantage in radiotherapy patients is that it can detect a decrease in myocardial strain function early before LVEF falls significantly [14]. Moreover, GLS has been recommended by The Task Force for cancer treatments and cardiovascular toxicity of the European Society of Cardiology (ESC) for the monitoring of chemotherapy-related cardiotoxicity [15]. Research by our group and others shows that GLS is more sensitive for the early detection of RIHD than LVEF and can detect RIHD early [8, 16]. In this study, 45 (41.7%) patients experienced early RIHD after pulmonary SBRT. The BACCARAT study also reported the incidence of early RIHD after general radiotherapy [17]. Their study included 79 breast cancer patients who did not receive chemotherapy. Heart damage was defined as a reduction of GLS ≥ 10% from baseline. Over 6-month follow-up after radiotherapy, 47% of patients suffered early RIHD. According to the consensus of ASE/EACVI on cancer therapeutic-related cardiac dysfunction, GLS is recommended for monitoring changes in cardiac function during cancer treatment. The consensus recommended that a reduction of more than 15% in GLS compared to baseline is likely to be of clinical significance and should be referred to a cardio-oncologist [18].

At present, the risk factors for heart damage caused by pulmonary SBRT have not been identified. Previous studies on the risk factors for RIHD caused by conventional radiotherapy have shown that the occurrence of RIHD is related to underlying cardiovascular disease, diabetes, the use of anthracycline drugs, cardiac dose, smoking and age [19, 20, 21]. In this study, we found that cardiac dose-related indices, the use of anthracycline drugs, hypertension, LVM, E/Em and left atrial pressure were associated with the occurrence of early RIHD. The maximum heart dose, the use of anthracycline drugs and hypertension were independent risk factors for early RIHD.

Comprehensive treatment involving chemotherapy, radiotherapy, targeted therapy and immunotherapy is the main treatment strategy for pulmonary tumors. However, almost all systemic cancer therapeutic drugs have cardiac toxicity [22, 23]. In patients with a history of antitumor therapy, the cardiac dose limitation during pulmonary SBRT is a clinical problem that needs to be solved at present. In this study, we analyzed the maximum heart dose for predicting early RIHD during SBRT in layers, further clarifying the maximum heart dose limit of pulmonary SBRT under different tumor treatment regimens, which can guide future clinical practice.

### Limitations

This study has some limitations. First, although it is the largest prospective cohort study to use GLS for the early detection of RIHD caused by SBRT, the sample is still relatively small, and it is a single-center study. These shortcomings may have biased the results. Our findings need to be verified by large-scale, multicenter, hard-endpoint clinical trials. Second, GLS was used to evaluate early RIHD after pulmonary SBRT in this study. Although its sensitivity is high, GLS is a subclinical cardiac injury index, and the relationship between GLS and long-term cardiovascular events cannot be determined. We will continue to expand the sample size, extend the follow-up time, and further observe the predictive value of GLS for long-term cardiovascular events. Radiotherapy can cause any structural damage to the heart, such as myocardium, valves, pericardium, and coronary arteries. Our current research only focuses on the left ventricle, and future studies will further explore the changes in other cardiac structures after pulmonary SBRT, which is of great significance for promoting a deeper understanding of RIHD.

## Conclusions

Among patients receiving pulmonary SBRT, the maximum heart dose of radiotherapy, the use of anthracycline drugs and hypertension are independently associated with the occurrence of early RIHD. These findings can be applied to predict early RIHD and screen for high-risk patients. Using appropriate cardiac limiting doses in patients with different tumor treatment backgrounds may improve the long-term prognosis of patients receiving pulmonary SBRT.

## Electronic supplementary material

Below is the link to the electronic supplementary material.

Supplementary Material 1

## Data Availability

Research data are stored in Peking University Third Hospital and will be shared upon request to the corresponding author.

## Abbreviations

2D-STE: Two-dimensional speckle tracking echocardiography
BED: Biologically effective doses
EQD2: Equivalent dose in 2-Gy fractions
GCS: Global circumferential strain
GLS: Global longitudinal strain
GRS: Global radial strain
GTV: Gross tumor volume
PTV: Planned target volume
RIHD: Radiation-induced heart damage
SBRT: Stereotactic body radiotherapy

## References

[1] Bergom C, Bradley JA, Ng AK, et al. Past, present, and future of Radiation-Induced cardiotoxicity: refinements in targeting, surveillance, and risk stratification. JACC CardioOncol. 2021;3(3):343–59. 34604796 10.1016/j.jaccao.2021.06.007 PMC8463722

[2] Lewis GD, Farach A. Cardiovascular toxicities of radiation therapy. Methodist Debakey Cardiovasc J. 2019;15(4):274–81. 31988688 10.14797/mdcj-15-4-274 PMC6977566

[3] Bradley JD, Paulus R, Komaki R, et al. Standard-dose versus high-dose conformal radiotherapy with concurrent and consolidation carboplatin plus Paclitaxel with or without cetuximab for patients with stage IIIA or IIIB non-small-cell lung cancer (RTOG 0617): a randomised, two-by-two factorial phase 3 study. Lancet Oncol. 2015;16(2):187–99. 25601342 10.1016/S1470-2045(14)71207-0 PMC4419359

[4] Chang HM, Okwuosa TM, Scarabelli T, et al. Cardiovascular complications of cancer therapy: best practices in diagnosis, prevention, and management: part 2. J Am Coll Cardiol. 2017;70(20):2552–65. 29145955 10.1016/j.jacc.2017.09.1095 PMC5825188

[5] Mitchell JD, Cehic DA, Morgia M, et al. Cardiovascular manifestations from therapeutic radiation: A multidisciplinary expert consensus statement from the international Cardio-Oncology society. JACC CardioOncol. 2021;3(3):360–80. 34604797 10.1016/j.jaccao.2021.06.003 PMC8463721

[6] Pjyc A, Prjm B, Lei F, et al. Stereotactic ablative radiotherapy for operable stage I non-small-cell lung cancer (revised STARS): long-term results of a single-arm, prospective trial with prespecified comparison to surgery. Lancet Oncol. 2021;22(10):1448–57. 34529930 10.1016/S1470-2045(21)00401-0 PMC8521627

[7] The Radiation Oncology Therapy Branch of the Chinese Medical Association, The Professional Committee of Cancer Radiotherapy of the Chinese Anticancer Association, Radiation Therapy Physicians Branch of the Chinese Medical Association. Chinese expert consensus on stereotactic radiotherapy for early Non-Small cell lung Cancer (2019 Edition). Chin J Oncol. 2020;42(7):522–30. 10.3760/cma.j.cn112152-20200116-00039 32842437

[8] Li T, Zhuang H, Wang Y, et al. Two-dimensional speckle tracking echocardiography in evaluating Radiation-induced heart damage. Asia Pac J Oncol Nurs. 2022;9(2):119–24. 35529415 10.1016/j.apjon.2021.12.008 PMC9072173

[9] Reshko LB, Kalman NS, Hugo GD, et al. Cardiac radiation dose distribution, cardiac events and mortality in early-stage lung cancer treated with stereotactic body radiation therapy (SBRT). J Thorac Dis. 2018;10(4):2346–56. 29850140 10.21037/jtd.2018.04.42 PMC5949491

[10] Cosset JM, Henry-Amar M, Girinski T, et al. Late toxicity of radiotherapy in Hodgkin’s disease. The role of fraction size. Acta Oncol. 1988;27(2):123–9. 3390343 10.3109/02841868809090332

[11] Joiner MC, Bentzen SM, Fractionation. The linear quadratic approach. Basic Clin Radiobiology. 2018;8:102–19.

[12] Tembhekar AR, Wright CL, Daly ME. Cardiac dose and survival after stereotactic body radiotherapy for Early-stage Non-Small-cell lung Cancer. Clin Lung Cancer. 2017;18:293–8. 28089158 10.1016/j.cllc.2016.12.007 PMC5413368

[13] Stam B, Peulen H, Guckenberger M, et al. Dose to heart substructures is associated with non-cancer death after SBRT in stage I-II NSCLC patients. Radiother Oncol. 2017;123(3):370–5. 28476219 10.1016/j.radonc.2017.04.017

[14] Lo Q, Hee L, Batumalai V, et al. Subclinical cardiac dysfunction detected by strain imaging during breast irradiation with persistent changes 6 weeks after treatment. Int J Radiat Oncol Biol Phys. 2015;92(2):268–76. 25968824 10.1016/j.ijrobp.2014.11.016

[15] Zamorano JL, Lancellotti P, Rodriguez MD, et al. 2016 ESC position paper on cancer treatments and cardiovascular toxicity developed under the auspices of the ESC committee for practice guidelines: the task force for cancer treatments and cardiovascular toxicity of the European society of cardiology (ESC). Eur J Heart Fail. 2016;37(36):2768–801. 10.1093/eurheartj/ehw211 27567406

[16] Skyttä T, Tuohinen S, Luukkaala T, et al. Adjuvant radiotherapy-induced cardiac changes among patients with early breast cancer: a three-year follow-up study. Acta Oncol. 2019;58(9):1250–8. 31219359 10.1080/0284186X.2019.1630751

[17] Walker V, Lairez O, Fondard O, et al. Early detection of subclinical left ventricular dysfunction after breast cancer radiation therapy using speckle-tracking echocardiography: association between cardiac exposure and longitudinal strain reduction (BACCARAT study). Radiat Oncol. 2019;14(1):204–14. 31727075 10.1186/s13014-019-1408-8 PMC6854785

[18] Plana JC, Galderisi M, Barac A, et al. Expert consensus for multimodality imaging evaluation of adult patients during and after cancer therapy: a report from the American society of echocardiography and the European association of cardiovascular imaging. J Am Soc Echocardiogr. 2014;27(9):911–39. 25172399 10.1016/j.echo.2014.07.012

[19] Galper SL, Yu JB, Mauch PM, et al. Clinically significant cardiac disease in patients with hodgkin lymphoma treated with mediastinal irradiation. Blood. 2011;117(2):412–8. 20858859 10.1182/blood-2010-06-291328

[20] Gaasch A, Schonecker S, Simonetto C, et al. Heart sparing radiotherapy in breast cancer: the importance of baseline cardiac risks. Radiat Oncol. 2020;15(1):117–26. 32448164 10.1186/s13014-020-01520-8 PMC7245801

[21] Lyon AR, López-Fernández T, Couch LS, Asteggiano R, Aznar MC, Bergler-Klein J, Boriani G, Cardinale D, Cordoba R, Cosyns B, Cutter DJ, de Azambuja E, de Boer RA, Dent SF, Farmakis D, Gevaert SA, Gorog DA, Herrmann J, Lenihan D, Moslehi J, Moura B, Salinger SS, Stephens R, Suter TM, Szmit S, Tamargo J, Thavendiranathan P, Tocchetti CG, van der Meer P, van der Pal HJH; ESC Scientific Document Group. 2022 ESC Guidelines on cardio-oncology developed in collaboration with the European Hematology Association (EHA), the European Society for Therapeutic Radiology and Oncology (ESTRO) and the International Cardio-Oncology Society (IC-OS). Eur Heart J. 2022; 43(41):4229–4361. 10.1093/eurheartj/ehac244 36017568

[22] Fanous I, Dillon P. Cancer treatment-related cardiac toxicity: prevention, assessment and management. Med Oncol. 2016;33(8):84. 27372782 10.1007/s12032-016-0801-5

[23] Vaflard P, Ederhy S, Torregrosa C, et al. [Fluoropyrimidines cardiac toxicity: 5-fluorouracil, capecitabine, compound S-1 and Trifluridine/tipiracil]. Bull Cancer. 2018;105(7–8):707–19. 29960638 10.1016/j.bulcan.2018.05.005

