# Supplementary material for: Risk factors for early radiation-induced heart damage in patients undergoing pulmonary SBRT
Source: Echo Res Pract. 2025 Jun 2;12:14. doi: 10.1186/s44156-025-00076-1 (PMC12128278; doi:10.1186/s44156-025-00076-1)
Supplement: Supplementary file 1 — Supplementary Material 1 [file 44156_2025_76_MOESM1_ESM.docx]

**Supplementary Appendix**

**eTable 1 Changes in cardiac damage indicators before and after SBRT.**

|  | Baseline  (n=108) | post-SBRT  (n=108) | p value |
| --- | --- | --- | --- |
| CK, U/L | 66.0 (43.5, 98.0) | 60.0 (43.0, 92.8) | 0.125 |
| CKMB, U/L | 9.0 (6.0, 13.0) | 9.0 (7.0, 12.8) | 0.708 |
| TnT, ng/mL | 0.008 (0.005, 0.012) | 0.009 (0.005, 0.013) | 0.070 |
| NT-proBNP, pg/mL | 76.0 (32.0, 121.5) | 69.5 (27.0, 147.0) | 0.961 |
| LAD, mm | 32.6±5.2 | 32.2±5.4 | 0.203 |
| LAA, cm^2^ | 17.0 (15.0, 19.0) | 16.0 (13.6, 19.5) | 0.351 |
| IVST, mm | 7.8 (7.1, 8.5) | 7.7 (7.0, 8.4) | 0.218 |
| LVEDD, mm | 45.8 (42.0, 48.0) | 45.2 (42.2, 48.9) | 0.311 |
| LVESD, mm | 27.7 (26.0, 30.0) | 27.0 (25.0, 30.0) | 0.056 |
| LVEF, % | 70.0 (67.0, 72.0) | 69.0 (67.0, 72.0) | 0.511 |
| LVM, g | 113.2±32.1 | 108.8±32.6 | 0.056 |
| E/A | 0.8 (0.7, 1.1) | 0.8 (0.7, 1.1) | 0.566 |
| E/Em | 6.0 (5.0, 8.0) | 6.0 (5.0, 7.4) | 0.434 |
| LAP, mmHg | 9.0 (8.0, 11.0) | 9.0 (8.0, 11.0) | 0.395 |
| GLS, % | -12.4±4.1 | -11.3±4.6 | <0.001* |
| GCS, % | -13.1±4.9 | -11.8±5.5 | 0.007* |
| GRS, % | 19.4±12.5 | 18.7±13.2 | 0.517 |

Values are median (interquartile range), or mean±SD. CK = creatine kinase; CKMB = creatine kinase-MB; TnT = troponin T; NT-proBNP = N-terminal pro-B-type natriuretic peptide; LAD = left atrial diameter; LAA = left atrial area; IVST = interventricular septum thickness at end-diastole; LVEDD = left ventricular end-diastolic dimension; LVESD = left ventricular end-systolic dimension; LVEF = left ventricular ejection fraction; LVM = left ventricular mass; E = early diastolic transmitral velocity; A = late diastolic transmitral velocity; Sm = systolic mitral annular velocity; Em = early diastolic mitral annular velocity; LAP = left atrial pressure; GLS = global longitudinal strain; GCS = global circumferential strain; GRS = global radial strain. *p < 0.05.

**eTable 2 Univariate Logistic regression analysis of early RIHD.**

|  | *B* | S.E. | Odds Ratio (95%CI) | p value |
| --- | --- | --- | --- | --- |
| Age, years | -0.003 | 0.008 | 0.997 (0.981, 1.014) | 0.728 |
| Gender, male, n (%) | -0.268 | 0.391 | 0.765 (0.355, 1.648) | 0.494 |
| Maximum heart dose, Gy | 0.060 | 0.014 | 1.062 (1.033, 1.092) | <0.001* |
| Hypertension, n (%) | 1.099 | 0.479 | 3.000 (1.173, 7.674) | 0.022* |
| Diabetes mellitus, n (%) | 0.720 | 0.580 | 2.054 (0.659, 6.400) | 0.214 |
| Coronary heart disease, n (%) | -0.188 | 0.758 | 0.829 (0.188, 3.660) | 0.804 |
| Anthracycline, n (%) | 1.030 | 0.420 | 2.800 (1.228, 6.383） | 0.014* |
| Targeted drugs, n (%) | 0.758 | 0.398 | 2.135 (0.979, 4.653) | 0.056 |
| Immunosuppressive agents, n (%) | 0.656 | 0.482 | 1.927 (0.749, 4.959) | 0.174 |
| ACEI/ARB, n (%) | 1.304 | 0.721 | 3.684 (0.897, 15.124) | 0.070 |
| β blockers, n (%) | 1.09 | 0.889 | 2.976 (0.521, 17.003) | 0.220 |
| statins, n (%) | -0.248 | 0.660 | 0.78 (0.214, 2.843) | 0.707 |
| smoking history, n (%) | -0.529 | 0.507 | 0.589 (0.218, 1.591) | 0.297 |
| TnT, ng/mL | 0.074 | 0.04 | 1.076 (0.995, 1.165) | 0.068 |
| NT-proBNP, pg/mL | 0.002 | 0.002 | 1.002 (0.998, 1.006) | 0.287 |
| LVM, g | -0.017 | 0.007 | 0.983 (0.970, 0.997） | 0.015* |
| LVEF, % | -0.054 | 0.05 | 0.947 (0.859, 1.044) | 0.273 |
| E/A | 0.146 | 0.526 | 1.157 (0.413, 3.242) | 0.781 |
| E/Em | 0.211 | 0.080 | 1.235 (1.056, 1.443) | 0.008* |
| left atrial pressure, mmHg | 0.165 | 0.063 | 1.179 (1.042, 1.336) | 0.009* |
| GLS, % | -0.024 | 0.051 | 0.977 (0.884, 1.079) | 0.640 |

RIHD = radiation-induced heart damage; ACEI = angiotensin converting enzyme inhibitor; ARB = angiotensin receptor blocker; TnT = troponin T; NT-proBNP = N-terminal pro-B-type natriuretic peptide; LVM = left ventricular mass; LVEF = left ventricular ejection fraction; E = early diastolic transmitral velocity; A = late diastolic transmitral velocity; Sm = systolic mitral annular velocity; Em = early diastolic mitral annular velocity; GLS = global longitudinal strain. *p < 0.05.
